# Endovascular Treatment of Intracranial Aneurysms

**DOI:** 10.3390/life11040335

**Published:** 2021-04-10

**Authors:** Antonis Adamou, Maria Alexandrou, Christian Roth, Achilles Chatziioannou, Panagiotis Papanagiotou

**Affiliations:** 1 Department of Radiology-Medical Imaging, Faculty of Medicine, University of Thessaly, University Hospital of Larissa, 41110 Larissa, Greece; antadamo@med.uth.gr; 2 Department of Diagnostic and Interventional Neuroradiology, Hospital Bremen-Mitte/Bremen-Ost, 28205 Bremen, Germany; mariazypern@hotmail.com (M.A.); christian.roth@me.com (C.R.); 3 First Department of Radiology, School of Medicine, National & Kapodistrian University of Athens, Areteion Hospital, 11528 Athens, Greece; achatzi@med.uoa.gr

**Keywords:** intracranial aneurysms, endovascular treatment modalities, coiling, flow diversion, flow disruption

## Abstract

Traditionally, surgical clipping was the only available treatment modality for intracranial aneurysms. However, in the last few decades, the endovascular therapy of intracranial aneurysms (IAs) has seen a tremendous evolution and development. From coiling to flow diversion and flow disruptor devices, endovascular treatment modalities have increased in number and received broader indications throughout the years. In this review article, the treatment modalities for the endovascular management of IAs are presented, emphasizing newer devices and technologies.

## 1. Introduction

In the last three decades, several treatment modalities for the management of intracranial aneurysms (IA) have been introduced. Consistent progress in material technology and innovation led to a constantly increasing number of novel applications used in the treatment of IAs. Additionally, indications for the endovascular treatment of IAs have gradually expanded. Currently, endovascular therapy has become the preferred treatment option compared to surgical clipping.

The first treatment modality introduced was coiling. After that, coiling-assistant devices were introduced, namely balloons and stents. These devices are associated with a better outcome; however, each device has specific limitations. At the start of the 21st century, flow diversion devices were introduced in the treatment of IAs, representing a novel stent with enhanced features compared to traditional stents. The most recent advancement in IA treatment modalities is flow disruption. Flow disruptors are mesh-braided devices deployed intrasaccularly, aiming to eliminate the limitations of stents and flow diverters.

The present review aims to highlight the main treatment modalities for the management of IAs. Furthermore, recent technological advances in coiling, with the use or absence of either stent or balloon assistance, flow diversion, and flow disruption are discussed.

## 2. Background of Intracranial Aneurysms

### 2.1. Epidemiology

In a population without comorbidities, the prevalence of unruptured intracranial aneurysms (UIAs) is estimated at 3.2%. The median age at diagnosis varies between studies, with an average of 49 years (20.5–76.9) [1]. However, studies involving younger patients (20.5–30.6) were performed primarily for disease screening rather than investigating the familial or comorbid nature of IAs. The estimated female and male prevalence ratio is 1.57. Additionally, the prevalence is significantly higher in individuals older than 30 years of age compared to those under 30 years. The prevalence of UIA in patients aged 30 years and older has been reported to vary between 3.6 and 6.5% [2].

Subarachnoid hemorrhage (SAH) incidence is estimated at 6 per 100,000 patient-years [3]. Ruptured IAs are 1.24 times more likely in women than in men [4], whereas rupture is also 2.1 times more likely in black individuals than white individuals [5]. Furthermore, smoking is an independent risk factor, with smokers at a higher risk of IA development and rupture, especially in individuals who started smoking at a young age [6].

### 2.2. Etiology

The polygenic nature of IA heritability is strongly suggested in the majority of the cases. Environmental risk factors include hypertension, smoking, alcohol consumption, and hypercholesterolemia. Autosomal dominant polycystic kidney disease (ADPKD), Ehlers–Danlos type IV syndrome, Marfan syndrome, neurofibromatosis type 1, and brain tumors are factors implicated in the comorbidity of the disease. Risk factors for aneurysmal rupture are female sex, age, black ethnicity, smoking, and an aneurysm size ≥7 mm in diameter [5,7]. Antiepileptic drugs and sex hormones have been shown to be associated with the formation of IAs [8]. In addition, the formation of IAs has been linked to genetic factors that promote smoking and high blood pressure, suggesting the overlap of genetic and environmental factors [8]. These give proof for epidemiologically identified risk factors.

Genetic factors have also been implicated, with an increased overall risk in patients with first- and second-degree relatives with IA or SAH. Multiple genetic loci have been investigated for a potential link with IA formation and/or rupture, including candidate polymorphisms located in the ANRIL (antisense non-coding RNA in the INK4 locus), SOX17, EDNRA (endothelin receptor type A), COL1A2 (collagen type I A2), COL3A1 (collagen type III A1), ACE (angiotensin-converting enzyme), IL-6 (interleukin 6), SERPINA3 (α1-antichymotrypsin), VCAN (versican), and HSPG2 (heparan sulfate proteoglycan 2) genomic regions [9].

### 2.3. Pathophysiology

The pathogenesis of IA is complex and not yet completely understood. In general, IAs are formed due to extracellular matrix defects or degradation, hemodynamic stress, and inflammatory response. In IAs, smooth muscle cells acquire a secretory rather than a contractile function. This feature contributes to the lack of elastin and collagen production and the secretion of matrix metalloproteinases (MMPs), which mediate extracellular matrix degradation and remodeling. Although not conclusive, smoking has been shown to decrease levels of α1-antitrypsin, the main inhibitor of MMPs [10]. The imbalance between MMPs and their inhibitors is believed to play a key role in IA formation. Other mechanisms induce elastin and collagen reduction, implicated in vessel wall defects and IA formation, such as lysyl oxidase copper deficiency [11].

Hemodynamic stress also induces MMP release by endothelial and smooth muscle cells and subsequent extracellular matrix degradation. Furthermore, it also causes mechanical endothelial damage, smooth muscle cell degeneration, and media thinning [12]. Inflammatory cell infiltration is also induced in the tunica media of the arterial wall. Mediators of the inflammatory response are the monocyte chemoattractant protein 1, NF-κB, angiotensin II, prostaglandin E2, prostaglandin E receptor subtype 2, IL-1β, IL-6, TNF-α, TLR4, and nitric oxide [13,14].

### 2.4. Classification

IAs can be classified based on multiple variables, including their etiology (congenital, acquired, dissecting, atherosclerotic, infectious, and tumorous), size (qualitative: micro, small, medium, large, giant, or supergiant; quantitative: size in mm), shape (saccular, fusiform, or mycotic), or anatomic location (internal carotid, anterior, middle, or posterior circulation) [15].

### 2.5. Anatomy

Most aneurysms are located in the Willis’s circle; however, distal aneurysms are located in branches beyond the circle of Willis. IAs are more frequently found on the medial cerebral artery (MCA), followed by the internal carotid artery (ICA), anterior cerebral artery (ACA), anterior communicating artery (AComA), posterior communicating artery (PComA), and vertebrobasilar arteries [1]. They are commonly small (<5 mm, 66%) in size, with only a small proportion (7%) larger than 10 mm [1]. The shape of IAs varies between saccular, fusiform, or mycotic. Bulge formation has been observed on the arterial wall, arterial bifurcations, or the basilar artery in fusiform IAs. The risk of rupture is higher in larger aneurysms, especially those more than 20 mm in diameter, and IAs located in the posterior circulation and the AComA.

### 2.6. Rupture Predicting Scores

Calculating the IA rupture risk can be somewhat challenging due to numerous risk factors, and the mechanisms behind rupture are usually complex. Several rupture predicting scores have been developed, namely the PHASES Score [16] and the Unruptured Intracranial Aneurysm Treatment Score (UIATS) [17], which calculate rupture scores according to clinical characteristics. The PHASES score calculates the risk of rupture based on the population (patient decent), hypertension, patient age, aneurysm size, the presence of earlier SAH from another aneurysm, and the aneurysm site.

Designed to help interventionalists decide whether to proceed with neurointervention or consider conservative management, the UIATS uses a more complex score. However, both the PHASES and UIATS scores exhibited low sensitivity in retrospective studies performed on SAH patients [18,19]. Recently, a third predicting score, the Intracranial Aneurysm Rupture Score (IARS), has been published [20].

## 3. Endovascular Management of IA

### 3.1. Detachable Coils

The main representative of coils is the historic Guglielmi detachable coil (GDC, Stryker Neurovascular) [21], which revolutionized interventional neuroradiology. The GDC was first introduced in 1990 and approved by the US Food and Drug Administration (FDA) in 1995. The electrolytic detachment mechanism of the GDC was designed to induce intra-aneurysmal thrombosis. Large case series confirmed the positive outcome of aneurysm coiling, with feasibility in 96.9% of ruptured aneurysms and 94.0% of unruptured aneurysms [22]. Furthermore, procedural mortality was 1.4% and 1.7% in ruptured and unruptured IAs, respectively. Morbidity rates were 8.6% in ruptured aneurysms and 7.7% in unruptured aneurysms. However, there was insufficient data on the intra-aneurysmal thrombotic effect of GDC, necessitating the need for the development of new materials with a longer likelihood of intra-aneurysmal thrombosis [23].

In general, coils are usually comprised of bare platinum. The procedure of IA sac packing consists of three stages: framing, filling, and finishing. Following the rapid technological advancement of the 21st century, new coils have been introduced with improved properties, leading to higher efficacy of aneurysm occlusions. The main alterations on the material aspects of the new coils were: (a) new innovative detachment methods, such as the use of the V-Grip detachment controller, which secures rapid coil detachment in 0.75 s; (b) the polymer cover of the coils, such as polylactic or polyglycolic acid microfilament (PGLA), hydrogel coating, or polypropylene stretch-resistant coating; (c) the coil shape, with either 2D helical, 3D, or complex shapes in framing, filling, and finishing coils; and (d) coil stiffness, either soft, supersoft, or nano-type coils. PGLA microfilament and hydrogel coating are reported to be more effective than bare platinum coils [24,25,26].

**Figure 1:**
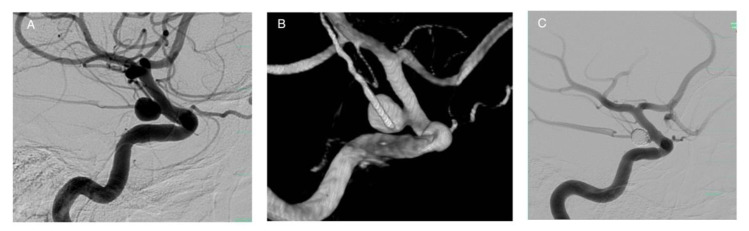
Stand-alone coiling of an ICA aneurysm with dome-to-neck ratios >2.0. The aneurysm is located at the supraclinoid segment of the right ICA (**A**,**B**), treated with detachable coils. Intraaneurysmal occlusion is visible on the image (**C**).

Stand-alone coiling is feasible for IAs with dome-to-neck ratios > 2.0 (Figure 1), excluding blood blister-like aneurysms, due to the wide neck, small size, and weak wall of these aneurysms, which pose a high risk of perforation [27]. Unassisted coiling is not recommended in large aneurysms with wide neck and low dome-to-neck ratio due to the high risk of poor outcomes [28]. Instead, balloon-assisted and stent-assisted coiling are two alternative methods that are considered safe, in respect of outcome, in complex aneurysms.

### 3.2. Balloon-Assisted Coiling (BAC)

**Figure 2:**
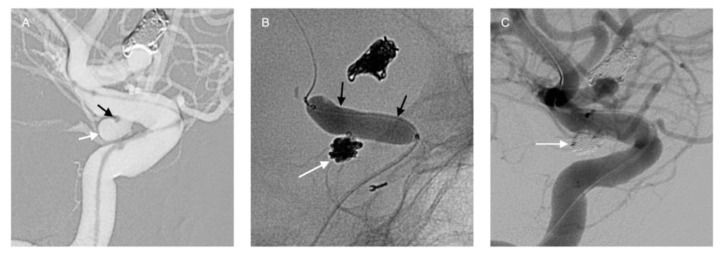
Balloon-assisted coiling of an ICA aneurysm (**A**, white arrow). The tip of the microcatheter within the aneurysm is seen (**A**, black arrow). The balloon is inflated in ICA (**B**, black arrows) while the coils in implanted within the aneurysm (**B**, white arrow). The successful occlusion of the aneurysm is shown in image (**C**, white arrow).

**Figure 3:**
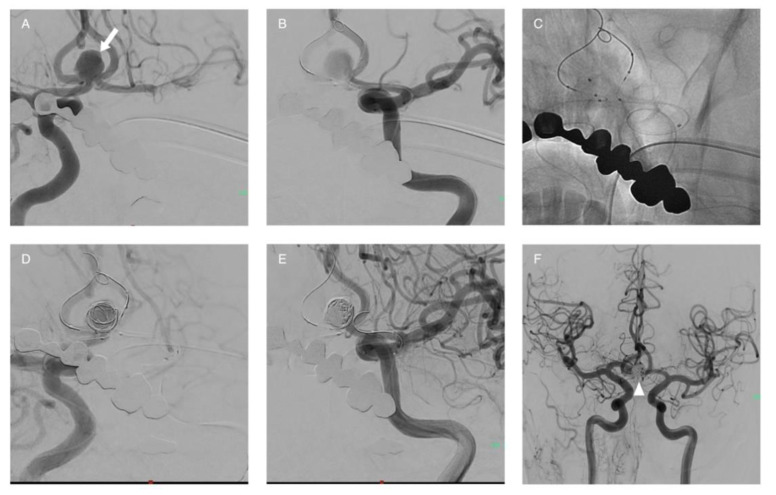
Double-balloon assisted coiling. Male patient with wide-neck saccular aneurysm located at the AComA (**A**, white arrow). Treated with detachable coils and simultaneous balloon protection in both ACAs (**B–D**). The successful occlusion of the aneurysm is shown in images (**E**,**F** white arrowhead).

The inflation of a balloon inside the parent artery is used in patients ineligible to undergo endovascular therapy with stand-alone coiling. The technique was first introduced by a French team [29] and is also known as the balloon remodeling technique. The balloon is placed across the IA neck, blocking the coil from potential intraprocedural displacement. The coil is deployed inside the aneurysm sac through a microcatheter that surpasses the balloon (Figure 2 and Figure 3). Therefore, the packing density of the coil within the aneurysmal sac may be higher [30]; however, there does not appear to be a decreased risk of a subsequent thromboembolic event due to coil displacement compared to stand-alone coiling [31]. Another complication associated with BAC is intraprocedural aneurysmal rupture. In the ATENA study [32], the rupture rate was higher in BAC patients (3.2%) compared to stand-alone coiling (2.2%). Sluzewski et al. [33] reported a more significant difference, with a rupture rate of 4% in BAC compared to 0.8% in unassisted coiling. However, another principal advantage of BAC is to control the bleeding in case of periprocedural rupture.

### 3.3. Stent-Assisted Coiling (SAC)

**Figure 4:**
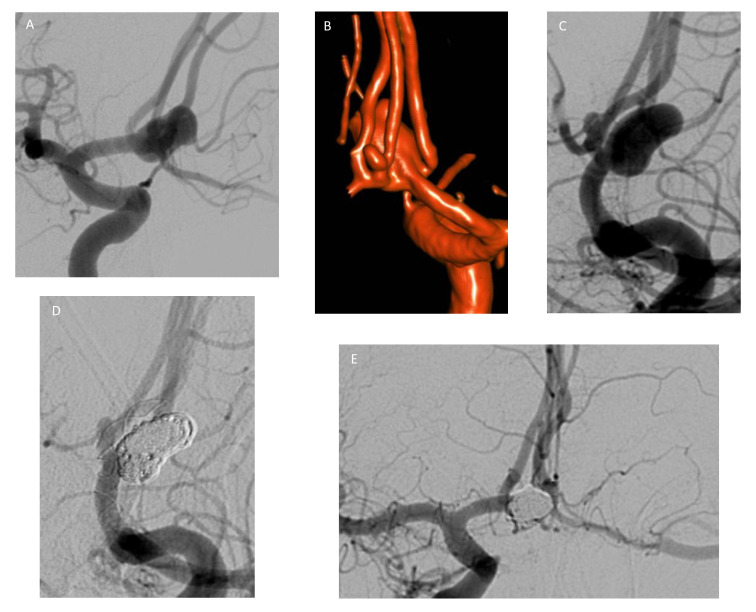
Stent-assisted coiling. Female patient with a complex wide-neck aneurysm of the AComA (**A**–**C**), three A2 branches arise from the neck. The implantation of a stent allowed to protect the branches and occlude the aneurysm completely (**D**,**E**).

Wide-neck aneurysms can be treated with a stent placement across the aneurysm entry point, as an addition to the coiling procedure. The catheter is passed through the stent openings, and the coil is deployed into the aneurysm. The use of stents rather than balloons has progressively been more widely adopted, especially for wide-neck complex aneurysms, to stabilize the coil mass inside the aneurysmal sac and avoid coil herniation into the parent artery [34,35]. At six-month follow-up, SAC offered a better outcome compared to BAC, with no statistical difference observed in retreatment rates and post-procedural complications [36] (Figure 4).

There are two types of stents: laser-cut nitinol stents and braided stents [37]. The foremost category is further subclassified into closed-cell and open-cell design stents.

### 3.4. Laser-Cut Stents

#### 3.4.1. Closed-Cell Stents

In closed-cell laser-cut stents, cells are closely attached, limiting the freedom of movement in each cell. This specification might cause bending, especially in curved arteries, forming a gap between the stent surface and the arterial wall [38]. The advantage of these stents is that they can be fully retracted, offering the possibility of resheathing.

#### 3.4.2. Open-Cell Stents

In open-cell stents, each cell has been designed to acquire a degree of freedom and, hence, better wall coverage. This type of stents can be used in arteries with high curvature, bifurcations, or arteries located in more distal locations.

#### 3.4.3. Advances

A variety of newer devices have been approved for use in Europe and/or the US. The majority of these stents were developed to confront complex, wide-neck, bifurcation aneurysms. Several studies have been conducted to assess their efficacy and safety to treat wide-neck bifurcation aneurysms, with favorable results concerning the outcome and safety.

#### 3.4.4. Braided Stents

Braided stents are nitinol stents that create a network of wires with closed loops on both ends. The design of these stents provides a better wall apposition, due to the wires’ ability to shorten or elongate each other, depending on the vessel curvature. This specification results in improved neck coverage in bifurcation aneurysms, since the stent wires shorten on the aneurysm neck portion and elongate on the opposite side, facilitating that blood flow surpasses uninterrupted.

The use of braided stents was investigated in multiple series [39,40,41,42,43,44,45,46,47]. The occlusion rates were 40–90% post-procedural, and 70–95% at follow-up have been suggested. Complication rates between 2% and 17% were also reported, as well as morbidity rates of 0–9% and mortality rates of 0–7%.

A recent meta-analysis [48] demonstrated complete occlusion of 54.6% post-procedural and 84.3% at follow-up, whereas the complication rate was 7%. Furthermore, the morbidity rate was 1.4%, with no cases of mortality. However, a noticeable rate of thromboembolic events was observed. Similar observations were reported in further studies [49,50,51]. Correspondingly, a novel stent has been examined for its safety and efficacy, with promising outcomes and satisfactory safety [52,53,54,55,56].

Further research is required to establish the possible superiority of braided stents over laser-cut stents and other devices.

### 3.5. Flow Diversion

Flow diverter devices were first introduced in 2007 in Europe, followed by the US four years later, owing to the need for an effective endovascular treatment option towards siphon-region, large (>10 mm), and wide-neck (>4 mm) aneurysms [57]. Flow diverters are indicated for aneurysms located in the internal carotid artery (ICA), from the petrous to the superior hypophyseal segments. Practically, flow diverter devices are stent-like, low-porosity metallic implants that are placed and left within the parent artery of IAs. Hence, all patients with a flow diverter in place require dual antiplatelet therapy.

The use of flow diverters becomes more frequent in patients with high intraoperative risk. It is the treatment of choice for most aneurysms of the posterior circulation, which tend to have a higher risk of rupture and compressive symptoms compared to the anterior circulation [58]. Additionally, in some cases where the standard surgical approach is contraindicated, flow diversion is an alternative treatment option. It also allows for staged treatment of multiple aneurysms, with favorable outcome and low morbidity and mortality rates [59].

**Figure 5:**
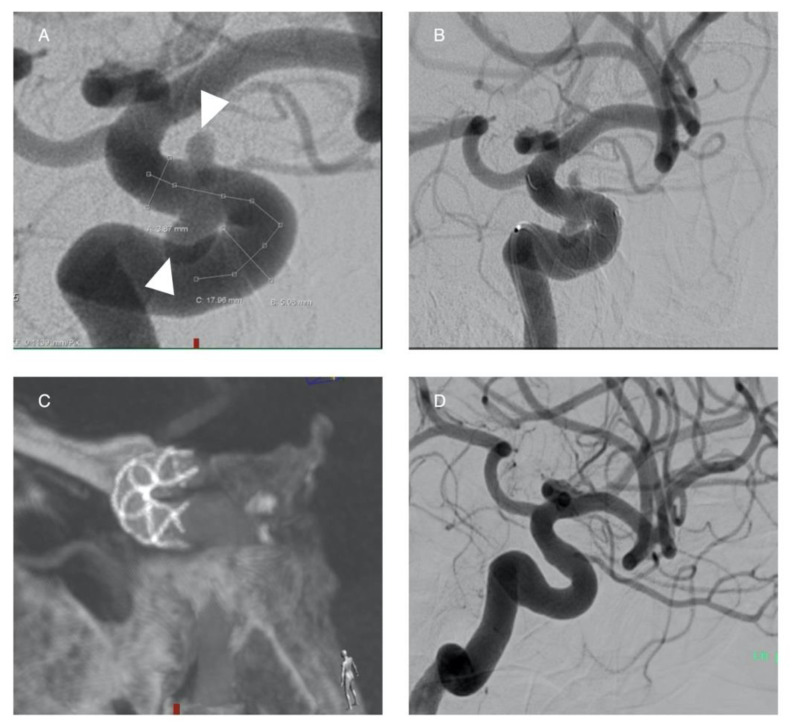
Female patient with multiple aneurysms (arrows) located at the supraclinoid segment of the left ICA (**A**). A Flow Diverter stent was deployed, covering both aneurysms (**B**,**C**). Postprocedurally, flow stagnation of the distal aneurysm is seen fully occluded (**B**). Follow-up angiography after six months shows complete occlusion of the aneurysms (**D**).

Flow diverters aim to decrease blood flow within the aneurysm and redirect the blood to the parent vessel. Low-porosity stents may lead to a reduction of up to 90% of the original flow inside the aneurysmal sac [60,61,62]. Additionally, flow diverters have a significantly higher surface coverage than traditional stents, promoting endothelial tissue formation along the surface of the metallic implant and subsequent permanent exclusion of the aneurysm from the systemic blood circulation [63,64] (Figure 5).

Newer devices have been examined in various trials, with differing degrees of efficacy. Notably, occlusion rates of 67–94% have been reported at six months follow-up, with morbidity rates ranging from 0–15%, and mortality rates between 0 and 5.5%, depending on the device and the study population [65,66,67,68,69,70,71,72,73,74,75,76,77,78,79,80]. Moreover, an occlusion rate of 100% was reported at three years follow-up [65,66,67,68,69,70,71,72,73,74,75,76,77,78,79,80,81]. However, further research is necessary to establish the consistency of these data.

**Figure 6:**
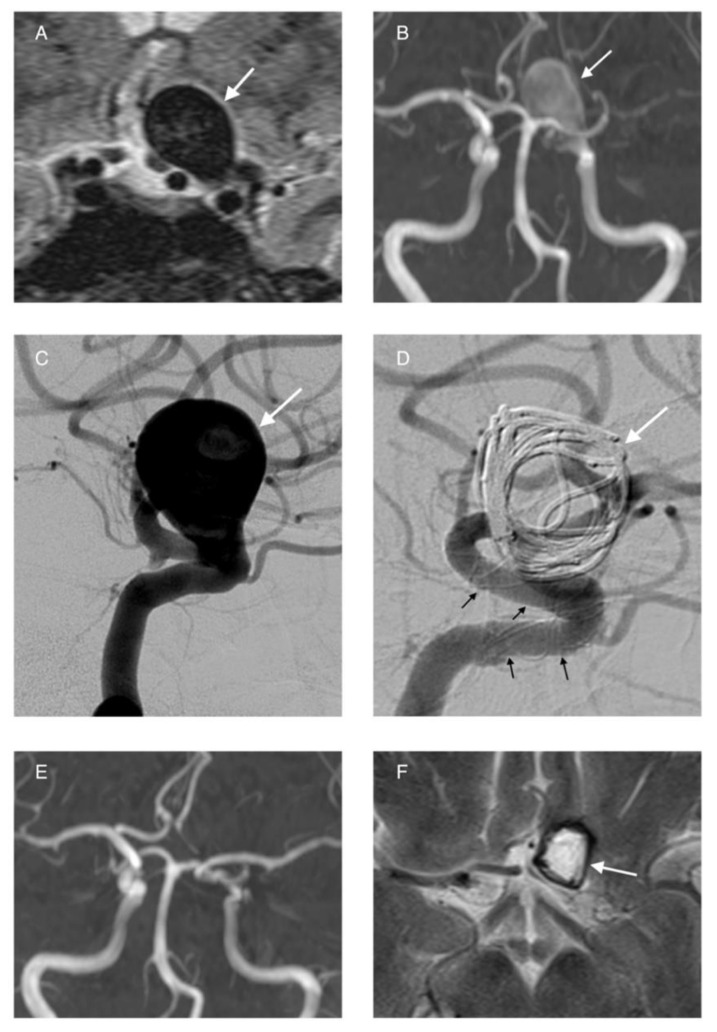
Giant aneurysm of the left ICA found on MRI and MR-angiography (**A**,**B**, white arrow) and clearly depicted with DSA (**C**, white arrow). The aneurysm was treated with implantation of a flow diverter (**D**, black arrows) and deposition of coils inside the aneurysm in order to induce thrombosis (**D**, with arrow). Follow up MRI and MRA show the completely occluded and thrombotic aneurysm (**E**,**F**, white arrow).

The proven efficacy of flow diverters has led to the expansion of indications for the treatment of complex or even non-complex IAs [82]. At present, flow diverters are indicated for the treatment of small- or medium-sized aneurysms [83,84,85,86,87] and fusiform aneurysms up to the ICA bifurcation, although the last indication remains challenging. Flow diverters can also be used especially in combination with coils in giant aneurysms (Figure 6).

Despite the treatment of distal aneurysms located beyond Willis’s circle considered challenging due to the narrow diameter of the vessels, it has now become feasible following the invention of finer catheters and more sophisticated devices. Some of these devices use a 0.021-inch microcatheter for their delivery, while others use an even finer microcatheter, at 0.017-inches. All devices are approved for use in Europe, with limited postmarket data assessing their efficacy [88].

Additionally, flow diverters have demonstrated favorable outcomes and low complication rates when used in the treatment of certain cases with complex, acutely ruptured aneurysms [89]. However, flow diversion devices should be used cautiously, and proper antithrombotic therapy must be considered to avoid the risk of recurrent hemorrhage [90]. At present, there are limited available data on durability of treatment results, therefore, further research is vital to investigate the safety and efficacy of flow diverters in the treatment of ruptured aneurysms.

### 3.6. Intrasaccular Flow Disruptors and Woven Endoluminal Bridge (WEB)

**Figure 7:**
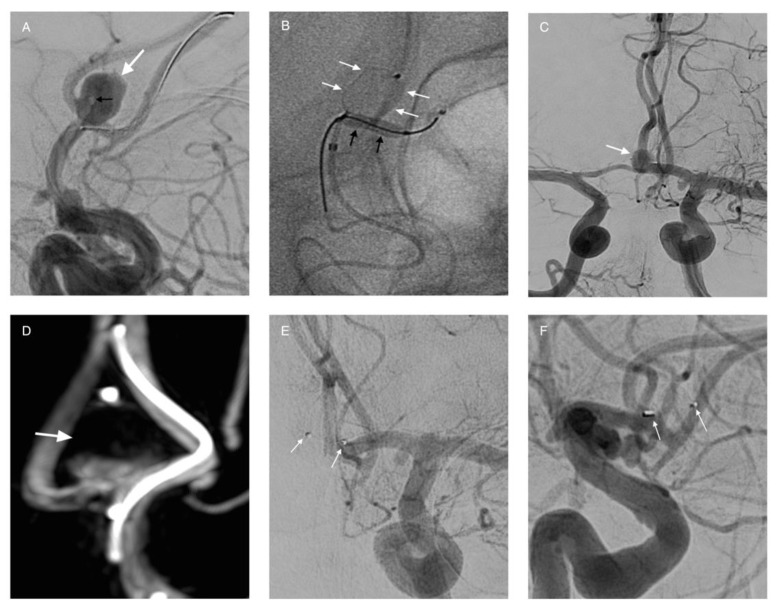
WEB-Device treatment. Male patient with a wide-neck aneurysm of the AcomA (**A**, white arrow). The tip of the microcatheter is seen within the aneurysm in order to place the WEBdevice (**A**, black arrow). The device was placed within the aneurysm (**B**, white arrows) assisted by a balloon for better adoption (**B**, black arrows). After placement of the device, DSA shows flow within the aneurysm (**C**, white arrow). However, flow stagnation of the upper part of the aneurysm is also seen (**D**, white arrow). Follow-up DSA after six months shows complete occlusion of the aneurysm, and only the markers of the WEB-device are visible (**E**,**F**, white arrows).

The development of flow disruptors was led owing to the risk of thromboembolic events with BAC and the risk of hemorrhage associated with antiplatelet therapy in SAC and flow diverters [91,92,93]. Flow disruptors are the most recent and sophisticated devices, developed to be used on overly wide-neck aneurysms and to overcome the limitations of previous devices. Similar to the flow diverter devices, they are also applicable in case of patients that surgery is not indicated. Technically, a flow disruptor is a metal mesh that is mounted within the aneurysmal sac, aiming to cause intra-aneurysmal thrombosis. Endosaccular deployment of the metallic mesh and the absence of a stent in the parent artery eliminates the risk [91,92,93] of intra-arterial thrombosis and the need for dual antiplatelet therapy in contrast to SAC and flow diversion (Figure 7).

The most studied flow disruptor is the WEB device, with results suggesting its safety for use in wide-neck bifurcation aneurysms. A recent meta-analysis [94], including 15 eligible studies, reported a feasibility rate of 96.7%, an occlusion rate of 83.3%, a retreatment rate of 8.4%, aneurysm perforation in 0.8%, and thromboembolic events in 5.6% of the treated aneurysms. These results indicate its potential safe use in aneurysms other than wide-neck bifurcation aneurysms. Mid-term occlusion rates are similar to those of SAC, and complication rates tend to be lower since antiplatelet therapy is not required.

Other than WEB, devices were also tested for their efficacy and safety. Occlusion rates were reported to be between 71% and 83% at follow-up, with noticeable recanalization rates (11–17%); however, data on morbidity and mortality were insufficient [95,96,97,98]. The European LUNA Aneurysm Embolization System (LUNA-AES) trial [99] reported occlusion rates of 88%, morbidity rates of 0% at 12 months and 1.8% at three years follow-up, and mortality rates of 1.6%, suggesting that the device is safe and effective for wide-neck bifurcation aneurysms. Further larger prospective studies are required to investigate their prospective versatility.

Despite these technologies holding promise for their independence from dual antiplatelet therapy, their efficacy and safety in terms of complications, morbidity, and mortality required investigation in more extensive, prospective studies.

## 4. Limitations

Research on novel materials for the endovascular treatment of IAs is ever increasing, therefore, the research work presented in the present review may not include all advancements in the field of intracranial techniques and devices.

## 5. Conclusions

Coil embolization with GDC or newer coils can be performed alone or with the combined use of balloons or stents. Complications owed to the use of stents and balloons, such as coil displacement and thrombosis, urged the need for newer approaches for the treatment of IAs. Novel materials are the flow diversion and the flow disruptor devices, which are confirmed for the treatment of siphon-located, large, or even wide-neck aneurysms. There are several available marketed devices. Among them, the WEB-device is a flow disruptor with promising results in terms of efficacy and complications. The durability and efficacy of novel devices remain subject to further research.

## Data Availability

Not applicable.
